# Usefulness of Cardiac MIBG Scintigraphy, Olfactory Testing and Substantia Nigra Hyperechogenicity as Additional Diagnostic Markers for Distinguishing between Parkinson’s Disease and Atypical Parkinsonian Syndromes

**DOI:** 10.1371/journal.pone.0165869

**Published:** 2016-11-03

**Authors:** Hiroaki Fujita, Keisuke Suzuki, Ayaka Numao, Yuji Watanabe, Tomoyuki Uchiyama, Tomoyuki Miyamoto, Masayuki Miyamoto, Koichi Hirata

**Affiliations:** 1 Department of Neurology, Dokkyo Medical University, Tochigi, Japan; 2 Continence Center, Dokkyo Medical University, Tochigi, Japan; 3 Department of Neurology, Dokkyo Medical University Koshigaya Hospital, Saitama, Japan; 4 Department of Clinical Medicine for Nursing, Dokkyo Medical University School of Nursing, Tochigi, Japan; Philadelphia VA Medical Center, UNITED STATES

## Abstract

**Background:**

We aimed to evaluate the utility of the combined use of cardiac ^123^I-metaiodobenzylguanidine (MIBG) scintigraphy, olfactory testing, and substantia nigra (SN) hyperechogenicity on transcranial sonography (TCS) in differentiating Parkinson’s disease (PD) from atypical parkinsonian syndromes (APSs), such as multiple system atrophy (MSA) and progressive supranuclear palsy (PSP).

**Methods:**

Cardiac MIBG scintigraphy, card-type odor identification testing (Open Essence (OE), Wako, Japan), and TCS were performed with 101 patients with PD and 38 patients with APSs (MSA and PSP). Receiver operating characteristic (ROC) curve analysis was used to assess the sensitivity and specificity of these batteries for diagnosing PD from APSs. The diagnostic accuracy of the three tests was also assessed among patients at the early disease stage (drug-naïve patients with a disease duration of 3 years or less).

**Results:**

In differentiating PD from APSs, the area under the ROC curve was 0.74 (95% CI, 0.65–0.83), 0.8 (95% CI, 0.73–0.87), and 0.75 (95% CI, 0.67–0.82) for TCS, cardiac MIBG scintigraphy, and olfactory testing, respectively. The diagnostic sensitivity and specificity were 53.1% and 91.7%, respectively, for TCS, 70.3% and 86.8%, respectively, for cardiac MIBG scintigraphy, 58.4% and 76.3%, respectively, for OE. Among early-stage patients, sensitivity and specificity were 50.0% and 93.8%, respectively, for TCS, 57.1% and 87.5%, respectively, for cardiac MIBG scintigraphy, and 54.8% and 79.2%, respectively, for OE. At least one positive result from 3 tests improved sensitivity (86.1%) but decreased specificity (63.2%). In contrast, at least 2 positive results from 3 tests had good discrimination for both early-stage patients (50.0% sensitivity and 93.8% specificity) and patients overall (57.8% sensitivity and 95.8% specificity). Positive results for all 3 tests yielded 100% specificity but low sensitivity (25%).

**Conclusions:**

At least 2 positive results from among TCS, cardiac MIBG scintigraphy, and olfactory testing can support clinical diagnosis in distinguishing PD from APSs.

## Introduction

Parkinson’s disease (PD) is a neurodegenerative disease that presents not only with cardinal motor features such as bradykinesia, rigidity and resting tremor but also with numerous non-motor signs. Despite recent advances in genetic and imaging studies, clinical diagnosis of PD based on neurological findings remains difficult, especially during the early stage of the disease. A clinicopathological study showed that for a disease duration of less than 5 years, only half of patients presenting with parkinsonism received a correct diagnosis of PD [1]. In a recent systematic review, the accuracy of a clinical diagnosis of PD made by movement disorders experts was found to be 80% [2]. Therefore, there has been increased awareness of the importance of assessing non-motor features, such as REM sleep behavior disorder, autonomic dysfunction and olfactory impairment, and several other clinical markers, including hyperechogenicity of the midbrain substantia nigra (SN), all of which can manifest during the early phase of the disease or even precede disease onset [3, 4].

Olfactory impairment occurs in 90% of early-stage PD patients and often antedates the onset of motor symptoms [5]. Thus, olfactory tests have been reported to be useful in differentiating PD from other atypical parkinsonian syndromes (APSs), such as multiple system atrophy (MSA) and progressive supranuclear palsy (PSP) [6–8].

The usefulness of cardiac ^123^I-metaiodobenzylguanidine (MIBG) scintigraphy, which assesses cardiac sympathetic nerve denervation, in distinguishing PD from APSs has been reported. Cardiac MIBG uptake is reduced in patients with PD, while its uptake is preserved in patients with MSA or PSP [7, 9, 10]. Olfactory loss and cardiac sympathetic denervation on MIBG scintigraphy have been included as supportive criteria in the updated MDS diagnostic criteria for PD [11].

In addition, hyperechogenicity of the SN on transcranial sonography (TCS) has been more frequently observed in patients with PD than in those with MSA or PSP [12, 13]. Taken together, these observations suggest that utilizing a combination of these batteries in addition to clinical assessment would be useful to achieve a correct diagnosis of PD. Only one study that used cardiac MIBG scintigraphy, olfactory tests and TCS showed that the combined use of these tests was useful in differentiating patients with PD from healthy controls [14]. However, no study has assessed the utility of the combination of these tests in distinguishing PD from APSs. In this study, we aimed to assess the utility of the combined use of cardiac ^123^I-MIBG uptake, olfactory function testing, and SN hyperechogenicity on TCS as additional diagnostic markers in differentiating PD from APSs such as MSA and PSP.

## Methods

This cross-sectional study was performed at the Department of Neurology, Dokkyo Medical University from April 2012 to March 2016. The study was approved by the institutional review board of Dokkyo Medical University Hospital and was conducted in accordance with the Declaration of Helsinki. All subjects enrolled in the study provided written informed consent.

### Subjects

**Fig 1:**
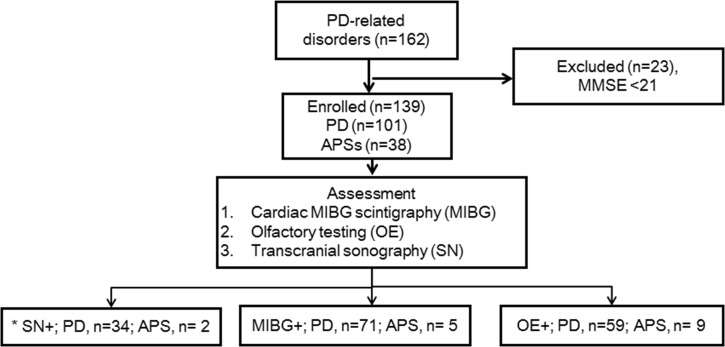
Flowchart of patient selection and the PD diagnostic accuracy of the combined use of cardiac MIBG scintigraphy, olfactory testing and transcranial sonography. * A total of 88 patients were included in the analysis (51 patients were not assessable for SN echogenicity by transcranial sonography). SN+ = substantia nigra hyperechogenicity positive; MIBG+ = cardiac MIBG scintigraphy positive; OE+ = olfactory testing positive.

Fig 1 shows a flowchart of patient selection. During the study period, 162 patients with parkinsonism were diagnosed with PD, MSA or PSP. After patients with cognitive dysfunction were excluded, 139 patients with PD-related disorders (101 PD patients, 21 MSA patients and 17 PSP patients) were included. The UK PD Society Brain Bank clinical diagnostic criteria were used for a diagnosis of PD [15]. Also, the established criteria were used for a diagnosis of MSA and PSP [16, 17]. Cases of secondary parkinsonism due to medication use or of parkinsonism related to vascular lesions or trauma were excluded. A clinical diagnosis of PD or APS among patients with parkinsonism was made, relying on clinical assessment by expert neurologists without knowledge of the results of TCS, olfactory testing or cardiac MIBG scintigraphy. Patients who scored 20 or less on the Mini-Mental State Examination (MMSE) were excluded. All the patients were followed for at least 3 years after disease onset to confirm the initial diagnosis. During follow-up, the clinical diagnosis of 1 patient was changed from PD to PSP, and that of another patient was changed from PSP to PD. Among the patients with MSA, 12 had MSA with predominant cerebellar ataxia (MSA-C), and 9 had MSA with predominant parkinsonism (MSA-P). Among the patients with PSP, 15 had Richardson’s syndrome, and 2 had PSP-parkinsonism. We analyzed subgroups of early-stage PD and APS patients, which were defined as drug-naïve patients with a disease duration of 3 years or less. For clinical assessment, disease severity was rated using the Hoehn and Yahr (HY) stage [18]. The levodopa equivalent dose (LED) was calculated according to previously reported methods [19].

### Transcranial sonography

TCS was performed using a conventional transcranial Doppler sonograph equipped with a 2.5 MHz transducer (LOGIQ 7; GE Healthcare, Tokyo, Japan). The midbrain was detected through bilateral temporal windows (6–8 cm). The transducer was set over the preauricular acoustic bone window, and the examination was carried out at a penetration depth of 14–16 cm and a dynamic range of 45–60 dB. An ultrasound unit was also used. The TCS was performed by two experienced examiners, KS and AN, who were blinded to the clinical diagnosis. The area of SN hyperechogenicity (cm^2^) was calculated from a frozen image that was expanded to 3- to 4-fold magnification [20]. When bilateral hyperechogenic SN areas were observed, the larger area was used for analysis. After patients with insufficient temporal bone windows were excluded, a total of 88 patients (63%) were included in the SN echogenicity analysis.

### Cardiac ^123^I-metaiodobenzylguanidine scintigraphy

The subjects were instructed to remain in a supine position for 15 minutes while 111 MBq ^123^I-MIBG (Fujifilm RI Pharma Co., Tokyo, Japan) was intravenously injected. Chest SPECT and planar images were obtained using a gamma camera 15 minutes (early phase) and 4 hours (delayed phase) after the injection. The heart-to-mediastinum (H/M) ratio was then calculated by dividing the count density of the left ventricular region of interest (ROI) by that of the mediastinal ROI, as described previously [21].

### Olfactory function

A card-type odor identification test (Open Essence (OE), Wako, Japan) was used. The OE test includes odorants compatible with those of the Odor Stick Identification Test for Japanese (OSIT-J). It can be performed more easily and more rapidly than the OSIT-J because it does not require the application, rubbing and handling steps required for the OSIT-J [22, 23]. The reliability of the OE test has been confirmed by its significant and strong correlation with the average detection and recognition thresholds of olfactometry [22]. The OE includes 12 different odors familiar to the Japanese population: India ink, wood, perfume, menthol, Japanese orange, curry, gas for household use, rose, Hinoki cypress, sweaty socks, condensed milk, and roasted garlic. During the test, when the subject opens the twice-folded card, a microcapsule breaks, and the odor is released. The subjects were asked to choose one of 6 possible answers: correct odor, odor closest to the correct one, odor close to the correct one, odor very different from the correct one, odor detectable but not recognizable, and no smell detected [22, 23]. A previous study found that an OE score ≤7 was useful for differentiating subjects with olfactory dysfunction from healthy volunteers [22]. In this study, the optimal cut-off score for the OE was set as 4 for differentiating PD from APS based on receiver operating characteristic curves

### Statistical analyses

A Mann-Whitney U-test or unpaired t-test was used, as appropriate, to compare continuous variables. A chi-square or Fisher's exact test was used to compare categorical variables between two groups. Based on receiver operating characteristic (ROC) curves, the sensitivity, specificity, positive predictive value (PPV) and negative predictive value (NPV) were calculated to determine optimal cut-off points of the diagnostic tools for differentiating PD from APSs. Spearman rank correlation coefficients were used to assess correlations. Statistical significance was defined as a two-tailed p<0.05. GraphPad Prism for Windows (Version 5.01; GraphPad Software, San Diego, USA) was used for the figures and ROC curve analyses, and IBM SPSS Statistics 22.0 (IBM SPSS, Tokyo, Japan) was used for the other statistical analyses.

## Results

The clinical characteristics of the patients with PD and APSs are shown in Table 1. There were no differences in mean age or in the distribution of the sexes between the groups. The patients with PD had a longer disease duration than the patients with APSs, whereas the disease severity was lower in the patients with PD than in those with APSs, according to the HY stages (2.7±0.9 vs. 3.0±0.7, p = 0.28). The percentage of de novo patients in the PD and APS groups was 50.1% and 68.4%, respectively. The delayed H/M ratio of cardiac ^123^I-MIBG uptake (1.9±0.1 vs. 2.8±0.6, p<0.001) and OE scores (3.9±2.5 vs. 6.2±2.4, p<0.001) were significantly lower in the patients with PD compared with the patients with APSs. The SN hyperechogenic area on TCS in the patients with PD was significantly larger than that in the patients with APSs (0.16±0.10 cm^2^ vs. 0.04±0.06 cm^2^, p<0.001). No significant differences in clinical background were observed between the total cohort (n = 139) and those patients with unsuccessful recording on TCS (n = 51) except for patient sex: the proportion of female patients was higher among patients with unsuccessful recording on TCS than among the total cohort.

**Table 1:** Demographic and clinical data of the study participants.

	PD (n = 101)	APS (n = 38) (MSA 21; PSP 17)	p-value
Age (years)	67.4±9.1	69.1±8.0	0.66
Sex (M/F)	47/54	14/24	0.31
Disease duration (years)	4.3±4.3	2.3±1.4	<0.001
HY stage	2.7±0.9	3.0±0.7	0.028
Cognitive function (MMSE)	27.3±2.7	26.5±2.8	0.12
LED (mg/day)	277.6±370.7	108.0±173.6	<0.001
Untreated patients, n (%)	51 (50.1)	26 (68.4)	0.058
Olfactory function (OE, Wako)	3.9±2.5	6.2±2.4	<0.001
MIBG delayed H/M ratio	1.9±0.1	2.8±0.6	<0.001
SN hyperechogenicity	0.16±0.1	0.04±0.06	<0.001

LED = levodopa equivalent dose; MIBG = ^123^I-metaiodobenzylguanidine, SN = substantia nigra

**Fig 2:**
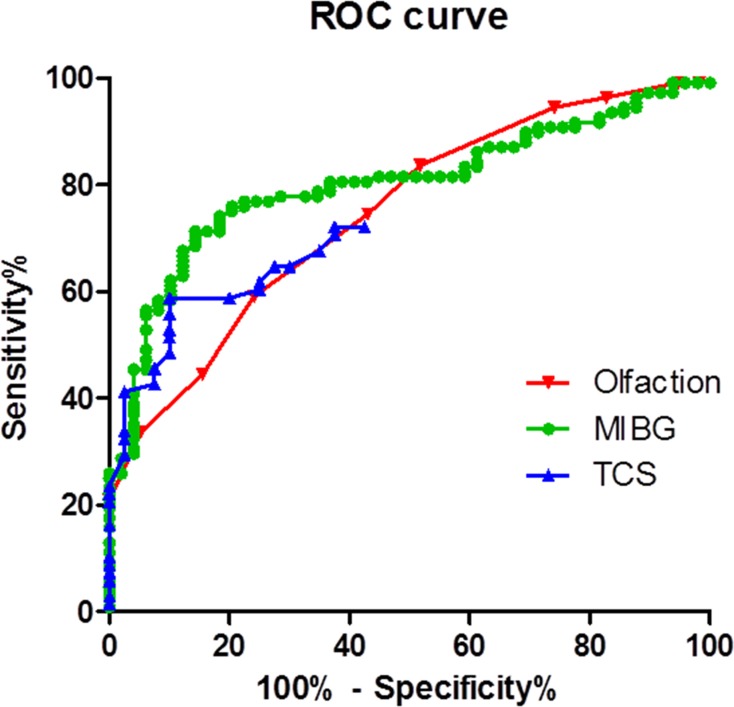
Receiver operating characteristic curves for the TCS, cardiac MIBG scintigraphy and olfactory function tests.

Fig 2 shows ROC curves for the TCS, cardiac MIBG scintigraphy and the olfactory function test for PD diagnosis. The area under the ROC curve (AUC) for cardiac MIBG scintigraphy, the olfactory function test, and SN hyperechogenic area on TCS in differentiating PD from APSs was 0.8 (95% CI, 0.73–0.87), p<0.001; 0.75 (95% CI, 0.67–0.82), p<0.001; and 0.74 (95% CI, 0.65–0.83), p<0.001, respectively. According to the ROC curve, we determined the optimal cut-off point for the measure of each diagnostic tool for differentiating PD from APSs as follows: cardiac MIBG scintigraphy (delayed H/M ratio) <2.00 (sensitivity 70.3%, specificity 86.8%); OE score ≤4 (sensitivity 58.4%, specificity 76.3%), SN hyperechogenic area on TCS ≥0.16 cm^2^ (sensitivity 53.1%, specificity 91.7%).

Table 2 illustrates the rate of positive TCS, cardiac MIBG and olfactory function test results in the PD and APS groups with different combinations of test batteries. Among all of the patients (Table 2A), each test had high specificity (76.3–91.7%) and high PPV (86.8–94.4%) in differentiation. Compared with the results from each test individually (option 1; TCS, MIBG or OE), at least one positive result among all 3 tests (option 2) improved the sensitivity of PD diagnosis but decreased the specificity. In contrast, at least 2 positive results among the 3 tests significantly improved the specificity and PPV of PD diagnosis (option 3; sensitivity, 59.4%; specificity, 94.7%; PPV, 96.8%). Positive results for all 3 tests (option 4) had 100% specificity and 100% PPV but low sensitivity (25%). In differentiating the PD group from the APS group, the subanalysis of early-stage patients (n = 40) showed similar trends as those observed for the total cohort for options 1–4, although in option 1, the sensitivity of a positive MIBG result was lower than that found in the analysis of all of the patients (Table 2B). As observed with the patients overall, among the early-stage patients, at least two positive results from 3 tests (option 3) had good discrimination (sensitivity, 50.0%; specificity, 95.8%; PPV, 95.5%). Within option 3, positive results from OE and positive results from either TCS or cardiac MIBG scintigraphy ((TCS or MIBG) and OE) had the highest specificity and the highest PPV for both the patients overall and the early-stage patients.

**Table 2:** Positive ratio of TCS, cardiac MIBG scintigraphy and olfactory function tests in the PD and APS groups.

**A)** Total patients (n = 139)								
**Combinations of test results**		**PD**	**APS**	**p-value**	**PD diagnosis**			
**option**					Sensitivity (%)	Specificity (%)	PPV (%)	NPV (%)
**1**	SN+ (≥0.16 cm2)	34 (53.1%)	2 (8.3%)	<0.001	53.1	91.7	94.4	42.3
	MIBG+ (≤2.0)	71 (70.3%)	5 (13.2%)	<0.001	70.3	86.8	93.4	52.4
	OE+ (≤4)	59 (58.4%)	9 (23.7%)	<0.001	58.4	76.3	86.8	40.8
**2**	At least one positive result from 3 tests	87 (86.1%)	14 (36.8%)	<0.001	86.1	63.2	86.1	63.2
**3**	At least two positive results from 3 tests	60 (59.4%)	2 (5.3%)	<0.001	59.4	94.7	96.8	46.8
	(SN or MIBG) and OE	52 (51.5%)	1 (2.6%)	<0.001	51.5	97.4	98.1	43
	(OE or MIBG) and SN	29 (45.3%)	1 (4.2%)	<0.001	45.3	95.8	96.7	39.7
	(OE or SN) and MIBG	56 (55.4%)	2 (5.3%)	<0.001	55.4	94.7	96.6	44.4
**4**	Positive results for all 3 tests	17 (26.6%)	0 (0%)	<0.001	26.6	100	100	33.8
**B)** Early-stage patients (n = 40)								
**Combinations of test results**		**PD**	**APS**	**p-value**	**PD diagnosis**			
**option**					Sensitivity (%)	Specificity (%)	PPV (%)	NPV (%)
**1**	SN+ (≥0.16 cm2)	12 (50.0%)	1 (6.3%)	0.004	50	93.8	92.3	55.6
	MIBG+ (≤2.0)	24 (57.1%)	3 (12.5%)	<0.001	57.1	87.5	88.9	53.8
	OE+ (≤4)	23 (54.8%)	5 (20.8%)	0.007	54.8	79.2	82.1	50
**2**	At least one positive result from 3 tests	32 (76.2%)	8 (33.3%)	0.001	76.2	66.7	80	61.5
**3**	At least two positive results from 3 tests	21 (50%)	1 (4.2%)	<0.001	50	95.8	95.5	52.3
	(SN or MIBG) and OE	20 (47.6%)	0 (0%)	<0.001	47.6	100	100	52.2
	(OE or MIBG) and SN	9 (37.5%)	1 (6.3%)	0.025	37.5	93.8	90	50
	(OE or SN) and MIBG	19 (45.2%)	1 (4.2%)	<0.001	45.2	95.8	95	51.7
**4**	Positive results for all 3 tests	6 (25.0%)	0 (0%)	0.03	25	100	100	47.1

SN+ = substantia nigra hyperechogenicity positive; MIBG+ = cardiac MIBG scintigraphy positive; OE+ = olfactory testing positive

Subanalyses showed the cardiac MIBG scintigraphy, OE and TCS tests were individually useful in differentiating the PD group from the PSP group and the PD group from the MSA group (Table 3).

**Table 3:** Positive ratios of TCS, cardiac MIBG scintigraphy and olfactory function tests for the PD vs. PSP and PD vs. MSA groups.

**(A)** PD vs. PSP								
**Combinations of test results**		**PD (n = 101)**	**PSP (n = 17)**	**p-value**	**PD diagnosis**			
**option**					Sensitivity (%)	Specificity (%)	PPV (%)	NPV (%)
**1**	SN+ (≥0.16 cm2)	34 (53.1%)	0 (0%)	0.003	53.1	100	100	23.1
	MIBG+ (≤2.0)	71 (70.3%)	2 (11.8%)	<0.001	70.3	88.2	97.3	33.3
	OE+ (≤4)	59 (58.4%)	5 (29.4%)	0.026	58.4	70.6	92.2	22.2
**2**	At least one positive result from 3 tests	87(86.1%)	7 (41.2%)	<0.001	86.1	58.8	92.6	41.7
**3**	At least two positive results from 3 tests	60 (59.4%)	0 (0%)	<0.001	59.4	100	100	29.3
	(SN or MIBG) and OE	52(51.5%)	0(0%)	<0.001	51.5	100	100	25.8
	(OE or MIBG) and SN	29(45.3%)	0(0%)	0.009	45.3	100	100	20.5
	(OE or SN) and MIBG	56(55.4%)	0(0%)	<0.001	55.4	100	100	27.4
**4**	Positive results for all 3 tests	17(26.6%)	0(0%)	0.078	26.6	100	100	16.1
(B) PD vs. MSA								
**Combinations of test results**		**PD (n = 101)**	**MSA (n = 21)**	**p-value**	**PD diagnosis**			
**option**					Sensitivity (%)	Specificity (%)	PPV (%)	NPV (%)
**1**	SN+ (≥0.16 cm2)	34 (53.1%)	2 (13.3%)	0.005	53.1	86.7	94.4	30.2
	MIBG+ (≤2.0)	71 (70.3%)	3 (14.3%)	<0.001	70.3	85.7	95.9	37.5
	OE+ (≤4)	59 (58.4%)	4 (19%)	0.001	58.4	81.0	93.7	28.8
**2**	At least one positive result from 3 tests	87(86.1%)	7(33.3%)	<0.001	86.1	66.7	92.6	50
**3**	At least two positive results from 3 tests	60(59.4%)	2(9.5%)	<0.001	59.4	90.5	96.8	31.7
	(SN or MIBG) and OE	52(51.5%)	1(4.8%)	<0.001	51.5	95.2	98.1	29.0
	(OE or MIBG) and SN	29(45.3)	1(6.7%)	0.006	45.3	93.3	96.7	28.6
	(OE or SN) and MIBG	56(55.4%)	2(9.5%)	<0.001	55.4	90.5	96.6	29.7
**4**	Positive results for all 3 tests	17(26.6%)	0(0%)	0.024	26.6	100	100	24.2

SN+ = substantia nigra hyperechogenicity positive; MIBG+ = cardiac MIBG scintigraphy positive; OE+ = olfactory testing positive; PPV = positive predictive value; NPV = negative predictive value.

The SN hyperechogenic area on TCS was weakly negatively correlated with MMSE and OE scores in the patients with PD but not in the patients with APSs (Table 4). In the patients with PD, the cardiac MIBG delayed H/M ratio was significantly positively correlated with OE (r = 0.51, p<0.001) and MMSE scores (r = 0.30, p = 0.002) and was negatively correlated with age and disease duration (r = -0.38, p<0.001 and r = -0.26, p = 0.009, respectively). In contrast, in the APS group, no significant correlation was observed between the cardiac MIBG delayed H/M ratio and the OE score or the MMSE score. The OE score was negatively correlated with age and positively correlated with the MMSE score in both the PD and APS groups.

**Table 4:** Correlation between the TCS test, olfactory test, and cardiac MIBG scintigraphy results and clinical parameters.

	TCS (SN hyperechogenicity)		Cardiac MIBG		Olfactory testing	
	PD	APS	PD	APS	PD	APS
**Cardiac MIBG**	-0.056	0.13				
**Olfactory testing**	-0.26*	-0.21	0.51**	0.12		
**Age**	0.098	0.088	-0.38**	-0.019	-0.24*	-0.51**
**MMSE**	-0.29*	-0.097	0.30**	0.14	0.28**	0.36*
**Disease duration**	0.20	-0.19	-0.26**	0.37*	-0.17	0.15
**HY stage**	0.20	-0.045	-0.19	0.24	-0.12	0.17

Spearman’s rank correlation coefficient

*p<0.05

**p<0.01

## Discussion

Our study is the first to show the usefulness of combining 3 different types of tests, specifically, cardiac ^123^I-MIBG uptake, olfactory function (OE) and SN hyperechogenicity on TCS tests, as additional diagnostic markers to aid in distinguishing between PD and APS patients. At least 2 positive results from among TCS, cardiac MIBG scintigraphy, and olfactory testing can support clinical diagnosis in distinguishing PD from APSs. Although TCS findings are not yet included in the updated MDS clinical diagnostic criteria for PD [11], cardiac MIBG scintigraphy, olfactory function and TCS might serve as early markers for premotor and prodromal PD [24–26]. Walter et al. [27] reported that 96% of PD patients showed SN hyperechogenicity on TCS, whereas only 9% of APS patients showed SN hyperechogenicity on TCS. Kikuchi et al. [7] reported that the sensitivity and specificity in discriminating PD patients from MSA patients were 85.71% and 76.20% for cardiac MIBG scintigraphy (with a H/M ratio cut-off value of 1.795) and 73.81% and 85.71% for olfactory testing. Reduced cardiac MIBG uptake and olfactory impairment assessed by the University of Pennsylvania Smell Identification Test has also been reported to assist the differential diagnosis between vascular parkinsonism and PD [28].

In the current study, although each test had high utility individually, different combinations of the 3 batteries performed differently in differentiating PD from APS. At least one positive result for the olfactory function test (OE score ≤4), cardiac MIBG scintigraphy (delayed H/M ratio <2.00) or SN hyperechogenicity (SN hyperechogenic area ≥0.16 cm^2^) showed high sensitivity but low specificity for the diagnosis of PD (Table 2A and 2B, option 2). In contrast, at least two positive results from among the 3 tests yielded good discrimination between PD and APS in both the total cohort and early-stage patients (Table 2A and 2B, option 3), suggesting the combined use of 2 out of 3 tests could support the clinical diagnosis of PD. Interestingly, within option 3, positive OE results in addition to either positive MIBG results or positive SN hyperechogenicity results provided good differentiation. Olfactory testing is easy to perform and can detect olfactory impairment, which is present in early-stage disease without limitation, except for patients with dementia. In contrast, cardiac MIBG scintigraphy may be particularly valuable considering that olfactory testing can be hindered by the presence of dementia or sinus diseases and that SN hyperechogenicity can be assessed in only a limited number of Japanese patients with PD. A positive result on all 3 tests had high specificity and PPV but low sensitivity for PD diagnosis (Table 2A and 2B, option 4).

In a previous study that included patients in the early stage of PD and MSA, cardiac MIBG scintigraphy was more sensitive in differentiating PD from MSA, while olfactory function tests had higher specificity [7]. In our study, cardiac MIBG scintigraphy showed higher sensitivity and specificity than the olfactory function test. Although 37% of patients could not be assessed by TCS due to insufficient temporal bone windows, SN hyperechogenicity showed higher specificity than cardiac MIBG scintigraphy or olfactory testing. A study of 65 PD patients conducted by Kajimoto et al. [29] found that at cut-off values of 0.16 cm^2^ for SN hyperechogenicity on TCS and 1.66 for cardiac MIBG scintigraphy, the rates of positive SN hyperechogenicity and cardiac MIBG scintigraphy were 80% and 73%, respectively. The combined use of TCS and cardiac MIBG scintigraphy showed a sensitivity of 97%. Izawa et al. [14] assessed the utility of the combined use of TCS, cardiac MIBG scintigraphy and olfactory testing in differentiating PD patients from healthy controls. They found that the positive results from either one or both tests in combination with SN hyperechogenicity and olfactory tests yielded a sensitivity of 91.2% and 47.1%, respectively, whereas positive results from either one or both tests combined with olfactory and cardiac MIBG scintigraphy tests yielded a sensitivity of 87.1% and 55.9%, respectively. The specificity of the combination of cardiac MIBG scintigraphy with either SN hyperechogenicity or olfactory testing was not assessed in that study. To date, the utility of combining these testing modalities in discriminating between patients with PD and patients with APSs has not been evaluated.

In patients with PD, olfactory impairment has been correlated with cardiac sympathetic denervation [30, 31], even in the early stages of the disease [32]. Lee et al. [33] reported that the degree of cardiac sympathetic dysfunction, as evaluated by cardiac MIBG scintigraphy, was positively correlated with the degree of olfactory identification impairment in patients with PD but not in patients with MSA. These findings are consistent with our study results and support the concept that olfactory impairment and cardiac sympathetic denervation might be potential indicators for Lewy pathology [34–36]. In previous work, the area of SN hyperechogenicity was found to be uncorrelated with cardiac MIBG scintigraphy or olfaction [14] and to remain stable over a mean follow-up of 6.4 years [37], suggesting that this area might be a stable marker of vulnerability in the nigrostriatal system [12]. Although SN hyperechogenicity did not predict motor progression, impaired uptake of cardiac MIBG scintigraphy and olfactory impairment may predict future motor and cognitive decline [38, 39]. Thus, adding SN hyperechogenicity testing, which has a different profile from cardiac MIBG scintigraphy and olfactory impairment, might offer additional support for the diagnosis of PD.

A limitation of this study is that patients with PSP and MSA, which are molecularly distinct disorders (tauopathies vs. synucleinopathies), were grouped together as APS patients. Although the subanalyses indicated that cardiac MIBG, OE and TCS tests were individually useful in differentiating PD from PSP and PD from MSA, these results should be confirmed by a study with a large sample size. In addition, the group sizes for some of the subgroup analyses were small, and a small number diagnostic misclassifications could have significantly impacted the overall findings. Another study limitation is that only 63% of the patients could be assessed for SN echogenicity by TCS due to insufficient temporal bone windows. The excluded group, with unsuccessful recording on TCS, contained a higher proportion of females than the included group, as observed in a previous study [40]. We believe the low rate of successful SN recording observed in this study may be due to ethnic differences between Caucasians and Asians. In the review by Berg et al. [41], SN echogenicity was not detectable in 5–10% of Caucasian individuals and in 15–60% of Asian individuals. Kajimoto et al.’s study of SN hyperechogenicity in Japanese PD patients [29] showed that the SN could be adequately viewed via TCS in 46.2% of 65 PD patients. Their rate of successful SN recording was similar to ours. In our study, TCS was performed by two experienced examiners who were blinded to the clinical diagnosis. Although technical difficulties may have partially contributed to these recording failures, we consider ethnic differences to have played a major role. Based on our study, in the case of TCS recording failure, the combined use of olfactory testing and cardiac MIBG scintigraphy may be a useful diagnostic tool for PD.

Dopamine transporter (DAT) scans are useful in distinguishing PD-related disorders from drug-induced parkinsonism, psychogenic parkinsonism, vascular parkinsonism and scans without evidence of dopaminergic deficit [42]. In our study, DAT scan data were not included, as DAT scanning has only recently been approved in Japan and has therefore been performed on a limited number of patients. However, a meta-analysis found that DAT scans cannot be used to distinguish between PD and APSs [43]. Umemura et al. [44] assessed the usefulness of the putaminal apparent diffusion coefficient (ADC) test and cardiac MIBG scintigraphy in differentiating PD and MSA-P patients. The results showed that both modalities were useful. Busse et al. [45] reported that the combined assessment of motor asymmetry, hyposmia and SN hyperechogenicity improved the diagnostic specificity for PD. Therefore, further studies of the differentiation of PD from APSs that include the putaminal ADC test and motor asymmetry would be interesting. In addition, our study may have included subjects with mild cognitive impairment; such impairment could have affected the olfactory testing.

In conclusion, the sensitivity and specificity of PD diagnosis increased by combining the use of two of three test results. Our study results confirmed the utility of the combined use of cardiac MIBG scintigraphy, SN hyperechogenicity on TCS and olfactory testing as diagnostic markers in distinguishing PD from APSs.
